# Supplementary material for: Chromothripsis during telomere crisis is independent of NHEJ, and consistent with a replicative origin
Source: Genome Res. 2019 May;29(5):737–49. doi: 10.1101/gr.240705.118 (PMC6499312; doi:10.1101/gr.240705.118)
Supplement: Supplemental Material [file supp_gr.240705.118_Supplemental_file_1.zip › contigs/annotated_contigs/DB53/contig.2.DB53_length_796_mean_cov_10.9246231156.docx]

**DB53_length_796_mean_cov_10.9246231156**

```
AGTCTATATAAAAAATACAAA|AAAAACAAATCAGTAGTCACTGTATAGCCTTATCCAGGACTAAGTAGGGTTCACATCTTAGAAAATA
                      >chr8:87813832-87814231 - E=3e-225
TCGCTTAAAATGAACTTTTCAAGCAGAAATATTCCTAGGCTAGGGATCATTTACTTGGCACAGAGAAAGACTTGAAATTTGGTCATGAA

AAATCTCACAAACCCACAGTCCCATTTACCCATACAAAACATTTAGACTTAAGGAAAAATAATCTCTGCATAGCATTTATTGCTATATT

CTAAGTCTGTATTCTAAAATATCTTTTTTATCTTGGACCAAAAATATTTTATGTAAATCAACCTTCTTTTATAGTGTCTTGAAAGGAAA

ATCTGATTCTGTTTATATAAATTTTCTTAGTCTGTTCTTCCAAGAAAGTATTCTTACACAGAATA|CAGAAAGTAAAGTGGTGGCTGAG
                                                                   >chr8:78941781-789421
CATGGTGGCTCACGCCTGTTAATCTCAGCACTTTGAGAGGCTGAGGCAGATGGATCATCTGAGGTCAGGAGTTTGAGGCCAGCCTGGCC
57 + E=2e-214
AACATGGTGAAACCCCGTCTCTACTAAAAATACAAAAATGAGCCGGGCCTGGTGATGCGTGCCTGTAATCCCAGCTACTCGGGAAGCTG

AGGCAGGAGAATCACTTGAACCCGGGAGGCAGAGGTTGCAGTGAGATGAGATCGCGCCATTGCACTCACTCCAGCCTGGGCAACAGAGC

GAGACTCCGTCTAAAAAAAAAAGTAGTGGTGAAATAGCTACTCCACCGACAATGTAGGGTGTTCCTGAAAGTAAGAGGAGGAATGC
```
